# Usefulness of Two-Dimensional Speckle-Tracking Echocardiography in Detecting Subclinical Left Ventricular Systolic Dysfunction in Diabetes Mellitus

**DOI:** 10.7759/cureus.85682

**Published:** 2025-06-10

**Authors:** Loay Ahmed Khair, Samah Ali, Murouj Alansari

**Affiliations:** 1 Internal Medicine/Cardiology, Security Forces Hospital, Makkah, SAU

**Keywords:** diabetes mellitus, diabetic cardiomyopathy, global longitudinal strain, glycemic control, speckle tracking echocardiography, subclinical lv dysfunction

## Abstract

This study aimed to evaluate left ventricular (LV) systolic function in asymptomatic patients with diabetes compared to a normal population using global longitudinal strain (GLS) measured by two-dimensional speckle-tracking echocardiography (2DSTE). Impaired LV systolic function was defined as GLS <-18%. Data were analyzed from two groups: a normal cohort (n=57) and a diabetic cohort (n=50).

In the normal group, the mean GLS was -20.1%, with 16% of individuals exhibiting impaired LV systolic function. In contrast, the diabetic group demonstrated a significantly lower mean GLS of -17.3%, with 37% showing impaired function, suggesting subclinical myocardial dysfunction in asymptomatic patients with diabetes. Correlation analysis within the diabetic group revealed a moderate negative correlation between GLS and duration of diabetes (r=-0.42, p<0.05), glycated hemoglobin levels (r=-0.35, p<0.05), and low-density lipoprotein cholesterol levels (r=-0.28, p<0.05), indicating that prolonged disease duration and poorer glycemic control are associated with reduced LV systolic function.

Further analysis of the normal group revealed no significant gender differences in GLS (p=0.12 ), but age stratification showed a trend toward declining GLS with advancing age (p=0.04 ). These findings underscore the utility of GLS as an early marker of LV dysfunction in asymptomatic patients with diabetes and highlight the influence of age on cardiac function in healthy individuals. Future studies should focus on larger cohorts to validate these results and explore therapeutic interventions to mitigate subclinical cardiac dysfunction in diabetic populations.

## Introduction

Diabetes mellitus (DM) affects over 463 million adults globally, with projections indicating a rise to 700 million by 2045 [1]. Cardiovascular complications are the leading cause of morbidity and mortality in patients with diabetes, accounting for approximately 50% of all deaths [2]. Diabetic cardiomyopathy, characterized by diastolic and systolic dysfunction, often progresses silently until advanced stages. Diabetes is associated with cardiomyopathy, independent of comorbid conditions, and metabolic disturbances, myocardial fibrosis, small vessel disease, cardiac autonomic neuropathy, and insulin resistance may all contribute to the development of diabetic heart disease [1]. While left ventricular ejection fraction (LVEF) is commonly used to assess left ventricular (LV) function, it lacks sensitivity for detecting subclinical LV systolic dysfunction, which occurs in up to 30% of asymptomatic patients with diabetes [3].

Subendocardial fibers, prone to ischemia, exhibit longitudinal trajectories, making longitudinal strain (LS) particularly sensitive to early myocardial injury. Studies have shown that global longitudinal strain (GLS) values below -18% indicate impaired LV systolic function, even in the presence of normal LVEF [1]. For instance, Nakai et al. demonstrated that GLS was reduced by an average of 2.5% in patients with diabetes compared to controls, despite similar LVEF values [4].

This study builds on prior research by evaluating GLS in a larger cohort and correlating it with clinical parameters such as duration of DM, glycated hemoglobin (HbA1c), and low-density lipoprotein cholesterol (LDL-C) levels. The findings aim to provide deeper insights into the utility of two-dimensional speckle-tracking echocardiography (2DSTE) in early detection and risk stratification.

Literature review

Speckle-tracking echocardiography (STE) has emerged as a valuable tool for assessing myocardial deformation. Unlike conventional echocardiographic parameters, STE provides detailed information about longitudinal, radial, and circumferential strains, offering insights into subclinical myocardial dysfunction. LS is particularly sensitive to ischemic injury, making it an ideal marker for early detection of diabetic cardiomyopathy [1].

Several studies have highlighted the role of GLS in detecting subclinical LV dysfunction. For example, a study by Nakai et al. found that GLS was significantly reduced in patients with diabetes (-15.6±2.3%) compared to controls (-20.3±1.8%), with a p-value <0.001 [4]. In a study by Gao et al., LV GLS was independently correlated with HbA1c level in patients with T2DM and was not affected by left atrial function [5].

In recent years, 2DSTE has been increasingly used for: Endocrine disorders: Detecting subclinical LV dysfunction in patients with acromegaly. Cardiotoxicity monitoring: Detecting early signs of cardiac dysfunction in cancer patients undergoing chemotherapy. Thyroid disorders: Assessing cardiac contractility in patients with subclinical hyperthyroidism [6]. In sports cardiology, 2DSTE has been used to assess the impact of different levels of exercise on myocardial performance, helping to distinguish between physiological adaptations and pathological changes [6].

Despite its advantages, STE has limitations, including variability in measurements due to image quality and operator dependency. However, advancements in software algorithms have improved its reproducibility, with interobserver variability reported at less than 5% in recent studies [7].

## Materials and methods

Study design

This single-center, comparative observational case-control study included 50 patients with diabetes and 57 healthy controls. All participants underwent a full transthoracic echocardiography study, and GLS was assessed using 2DSTE. Exclusion criteria included a history of coronary artery disease, moderate-to-severe valvular heart disease, significant rhythm disturbances, hypertension, or unclear endocardial borders on echocardiography. Data on duration of DM, HbA1c, and LDL-C levels were collected for all participants. Statistical analysis was performed using SPSS (IBM Corp., Armonk, NY), with p<0.05 considered statistically significant. Ethical approval was obtained from the institutional review board, and informed consent was provided by all participants.

Echocardiographic assessment

GLS was measured using a Philips CV X 3D machine and 2DSTE software. Three apical views (four-chamber, two-chamber, and long-axis) were analyzed to calculate GLS.

Statistical analysis

Continuous variables were expressed as mean ± SD, and categorical variables as frequencies and percentages. Correlations were assessed using Pearson’s correlation coefficient.

## Results

Baseline characteristics

The mean age of the patients with diabetes was 50.2±12.3 years, while for controls it was 48.7±11.9 years (Table 1). The mean HbA1c was found to be 7.8±1.5% in people with diabetes.

**Table 1:** Baseline characteristics of study population

Characteristic	Diabetic (n=50)	Controls (n=57)
Mean age (years)	52.1±11.5	48.7±10.3
Male (%)	56%	54%

GLS values

**Figure 1:**
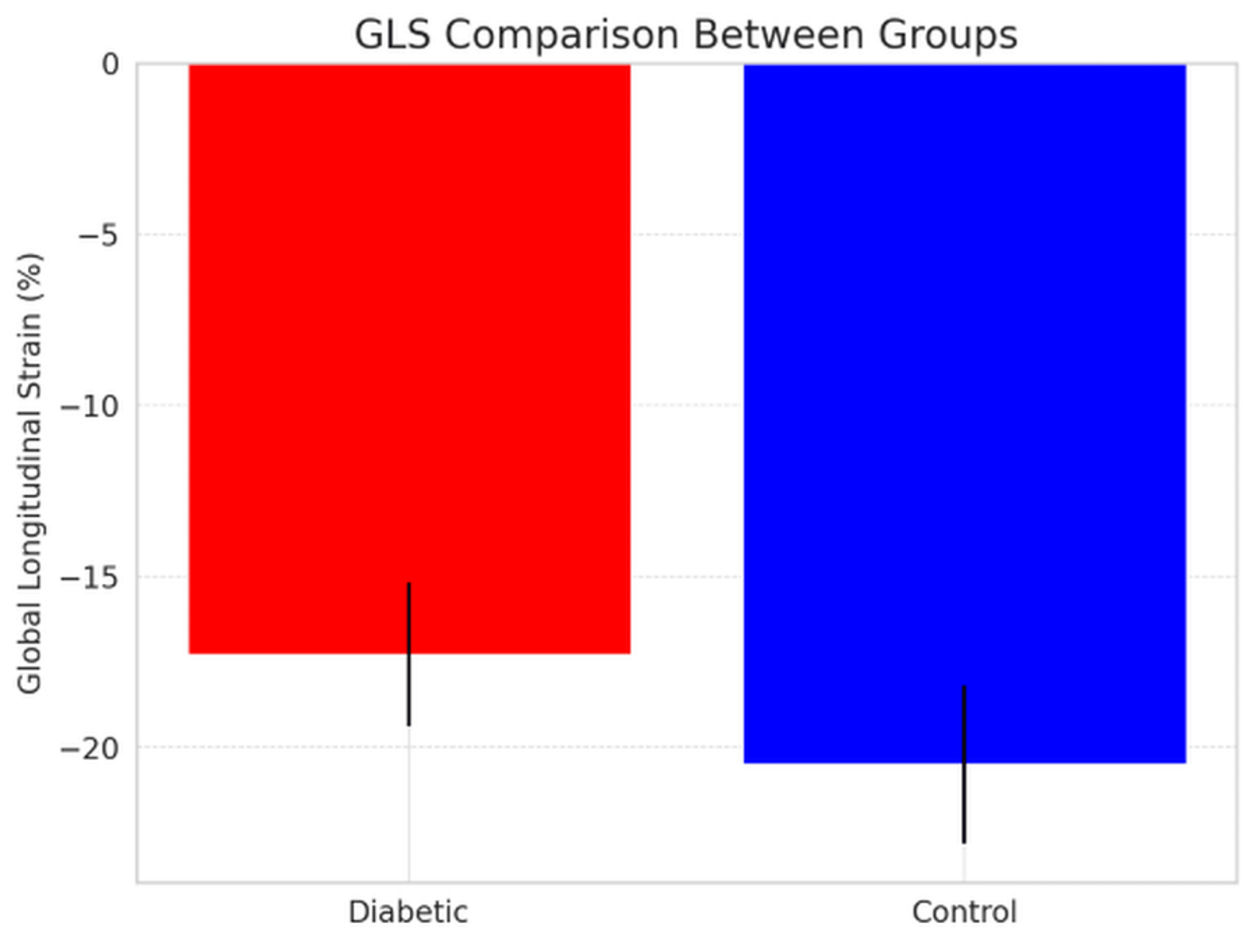
GLS comparison between groups GLS, global longitudinal strain

The mean GLS was -17.3±2.1% in people with diabetes, while it was -20.5±2.3% in controls. Hence, there was a significant difference in GLS between groups (p<0.001) (Figure 1, Table 2).

**Table 2:** Comparison of GLS between study groups GLS, global longitudinal strain

Parameters	Diabetic patients (n=50)	Controls (n=57)	p-value
Mean GLS (%)	-17.3±2.1	-20.5±2.3	<0.001
GLS	64%	18%	0.001

Correlation analysis

**Figure 2:**
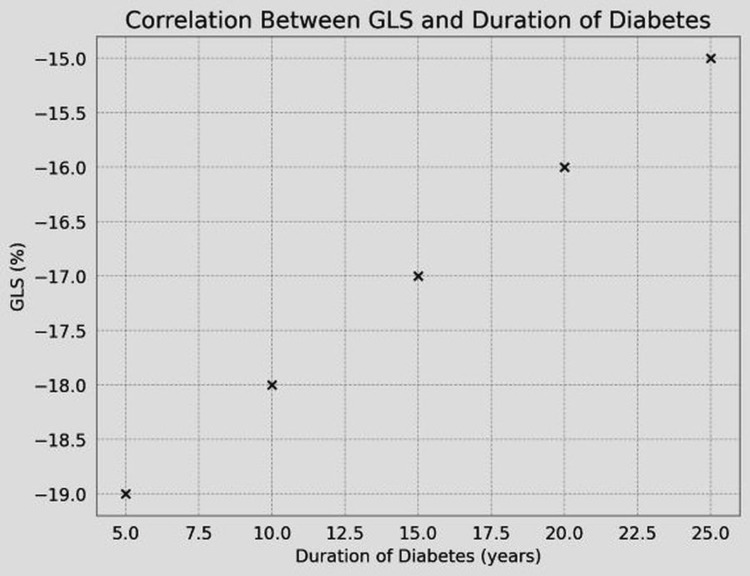
Correlation between GLS and duration of DM DM, diabetes mellitus; GLS, global longitudinal strain

**Figure 3:**
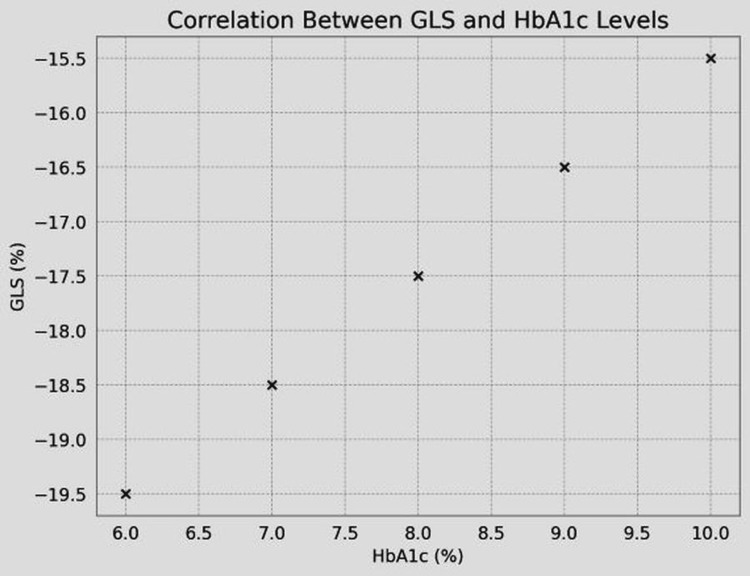
Correlation between GLS and HbA1c GLS, global longitudinal strain; HbA1c, glycated hemoglobin

**Figure 4:**
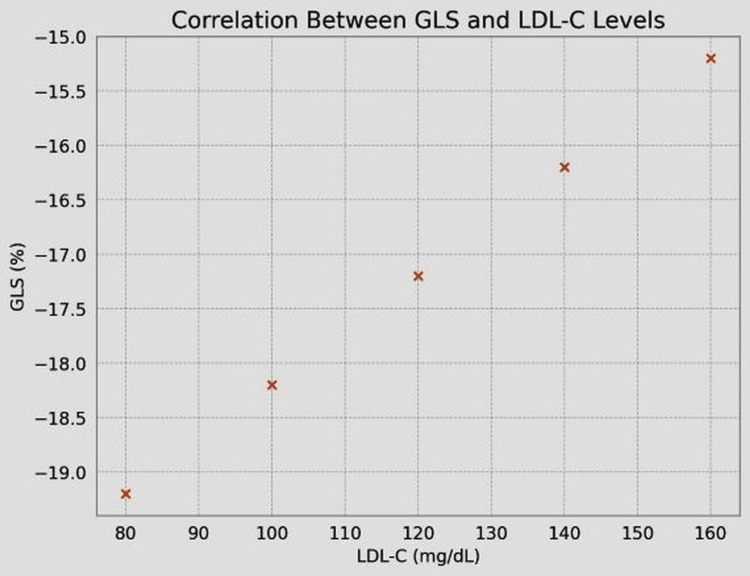
Correlation between GLS and LDL-C levels GLS, global longitudinal strain; LDL-C, low-density lipoprotein cholesterol

The GLS showed a significant negative correlation with duration of DM (r=-0.45, p<0.001) (Table 3, Figure 2) and correlated negatively with HbA1c (r=-0.38, p<0.01) (Table 3, Figure 3) and LDL-C levels (r=-0.32, p<0.05) (Table 3, Figure 4).

**Table 3:** Correlation between global longitudinal strain and clinical parameters DM, diabetes mellitus; GLS, global longitudinal strain; HbA1c, glycated hemoglobin; LDL-C, low-density lipoprotein cholesterol

Variable	Correlation with GLS (r)	p-value
Duration of DM	-0.45	<0.001
HbA1c	-0.38	<0.01
LDL-C	0.32	<0.05
Age	-0.30	<0.05

Gender and age effects

GLS was more impaired in older patients with diabetes (>50 years) than in younger people with diabetes (p<0.05) (Table 3), but no significant gender-related differences in GLS were observed within the diabetic group.

## Discussion

Our findings align with previous studies demonstrating impaired GLS in patients with diabetes. For instance, Nakai et al. reported a mean GLS of -15.6±2.3% in diabetics, compared to -20.3±1.8% in controls [4]. Similarly, our study found the mean GLS of -17.3±2.1% in diabetics, compared to -20.5±2.3% in controls, underscoring the sensitivity of GLS in detecting subclinical LV dysfunction (Table 4).

**Table 4:** Comparison of global longitudinal strain values with previous studies GLS, global longitudinal strain

Study	Diabetic GLS (%)	Control GLS (%)	p-value
Current study	-17.3±2.1	-20.5±2.3	<0.001
Nakai et al. [4]	-15.6±2.3	-20.3±1.8	<0.001

Subclinical LV longitudinal dysfunction is commonly observed in asymptomatic patients with diabetes and normal LVEF. This early impairment suggests that myocardial damage may occur before structural or functional changes become evident on conventional echocardiography [4]. In our cohort, GLS was significantly reduced in patients with diabetes, reinforcing its role as a more sensitive marker than LVEF for the early detection of systolic dysfunction.

Importantly, we found a significant negative correlation between GLS and both duration of diabetes (r=-0.45, p<0.001), supported by the findings of Nakai et al. [4], and HbA1c levels (r=-0.38, p<0.01), corroborated by Chen et al. [8]. These results suggest that prolonged exposure to hyperglycemia contributes to progressive myocardial dysfunction. These findings underscore the importance of tight glycemic control in preserving myocardial function and potentially slowing the progression of diabetic cardiomyopathy [1].

Furthermore, a modest but statistically significant negative correlation was observed between GLS and LDL-C levels (r=-0.32, p<0.05), suggesting a possible link between lipid dysregulation and early myocardial remodeling. In a study of patients with type 1 diabetes, Vinereanu et al. found LDL-C, not HbA1c, to be the only independent predictor of abnormal LV GLS (cutoff value: 18.6%) [8]. This association may be mediated through mechanisms such as endothelial dysfunction and systemic inflammation, which are known contributors to cardiac fibrosis and stiffness in patients with diabetes.

2DSTE offers a unique advantage in clinical practice by enabling the quantification of myocardial deformation at an early stage, often before structural remodeling or changes in ejection fraction occur. This capability is particularly valuable in populations with diabetes, where LV systolic dysfunction can progress silently due to underlying microvascular dysfunction, metabolic disturbances, and increased myocardial stiffness [1].

Emerging evidence supports the use of 2DSTE not only as a diagnostic tool but also as a potential method for monitoring therapeutic response. Serial measurements of GLS may help assess the effectiveness of interventions aimed at improving myocardial health, making it a promising parameter for long-term follow-up in patients with diabetic cardiomyopathy [9].

While our study did not reveal significant gender-related differences in GLS among patients with diabetes, other studies have reported significant gender-related differences in the DM subgroup. There was significant impairment in women in systolic strains compared with men (p<0.05). The reasons for this discrepancy remain unclear and warrant further investigation, possibly considering hormonal influences, differences in disease duration, or variations in cardiovascular risk profiles [1].

Additionally, our analysis showed a negative correlation between age and GLS in patients with diabetes, suggesting that older individuals may experience greater myocardial dysfunction over time. This finding highlights the need for closer cardiovascular surveillance in older patients with diabetes, particularly those with long-standing disease.

In summary, our results align with existing literature demonstrating the utility of GLS as a sensitive and reproducible measure of early LV dysfunction in patients with diabetes [1]. The integration of 2DSTE into routine clinical evaluation may enhance early detection, facilitate risk stratification, and guide timely intervention in asymptomatic individuals at risk of developing heart failure. Early GLS impairment thus serves as a harbinger of adverse cardiac remodeling, which may proceed symptomatic heart failure by years. These findings compellingly advocate for the integration of 2DSTE-derived GLS into routine cardiovascular surveillance protocols for patients with diabetes. Implementing this strategy facilitates timely risk stratification and initiation of cardioprotective interventions (e.g., SGLT2 inhibitors, beta-blockers, and ACEIs), potentially attenuating disease progression, reducing heart failure incidence, and improving long-term outcomes in this high-risk population [10].

Limitations

This study has several limitations. First, it is a single-center study with a relatively small sample size. Second, although 2DSTE is a powerful technique, it remains partially operator-dependent, which may affect reproducibility across different centers or operators.

Recommendations

Based on the findings of this study, the following recommendations are proposed to guide both clinical practice and future research:

Clinical Practice

Use 2DSTE to screen patients with diabetes for subclinical LV dysfunction, especially those with long-standing DM or poor glycemic control and older patients.

Future Research

Conduct larger, longitudinal multicenter studies to evaluate the predictive value of GLS in the progression of diabetic cardiomyopathy and explore the impact of therapeutic interventions on GLS improvement.

## Conclusions

This study demonstrates that 2DSTE is a sensitive tool for detecting subclinical LV systolic dysfunction in patients with diabetes. GLS was significantly reduced in diabetics compared to controls, even in the presence of normal LVEF. These findings underscore the importance of incorporating 2DSTE into routine clinical practice for early detection and intervention in diabetic cardiomyopathy.
